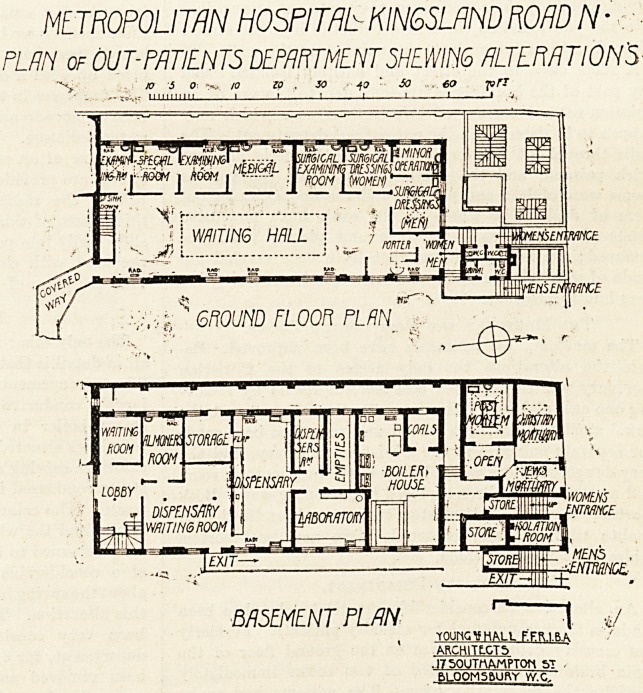# Metropolitan Hospital Reconstruction

**Published:** 1910-03-12

**Authors:** J. Courtney Buchanan


### METROPOLITAN HOSPITAL RECONSTRUCTION.

By J. COURTNEY BUCHANAN.

On November 15 last this hospital was re-opened for the reception of patients after having been closed for rather more than four months. During this period extensive works in the nature of alterations, improvements, and much-needed repair have been carried out under the direction of Messrs. Keith D. Young and Henry Hall, F.F.R.I.B.A., architects, 17 Southampton Street, Bloomsbury, W.C.

The hospital was built in 1885, and the reconstruction which has just been completed was rendered necessary by lapse of time and the advance of knowledge.

The walls of the wards and offices, staircases, and passages were originally left in bare brickwork and painted. The ward floors are all laid with oak and are in good condition; but the floors of the sanitary offices were laid with cement, which was in a very bad condition; and in the ward kitchens the floors were of deal, and that in a bad state. The brick walls throughout have been plastered and painted; the cement and deal floors have been replaced by Terrano flooring.

### Sanitary Fittings.

The whole of the sanitary fittings, which were of an obsolete type throughout, and in many cases worn out, have been replaced by the best known appliances of their several kinds. In particular we would mention the baths, which are made to a special pattern suggested by the architects, in which the dirt-collecting space underneath the bath is entirely avoided by making the outer wall of the bath continue down to the floor with a well rounded sweep. The baths in each case are fixed a sufficient distance away from the walls on both sides to allow of easy access all round, both for dealing with a helpless patient and for cleaning.

### Out-patient Department.

Perhaps the most important structural alteration is that which concerns the out-patient department. Before the alterations took place the out-patient department (which is in a separate two-story building isolated from the rest of the hospital) was arranged as follows. There were two separate entrances from the street for men and women, and separate waiting-rooms on the ground floor. Commanding both entrances was a room for the porter for the distribution of letters and so forth. From these two waiting-rooms patients passed into a passage, on either side of which were arranged six consulting-rooms. These six rooms had to serve all purposes for physicians, surgeons, and the special departments—Jewish, eye, throat and ear, dental, and diseases of women. From the consulting-rooms patients passed by two staircases, one for each sex, down into the basement, where they were interviewed by the almoner, after which they obtained their medicines at two different hatches in the dispensary, and left by two separate doors. In the department as it now stands the division of the patients into male and female is entirely done away with. The two entrances are retained because the sanitary offices are placed at the entrances; but, when the patients enter the hall, they are no longer separated by barriers. At the entrance is a desk, with a screen for the porter, and a turnstile to control the entrance of patients. The patients pass into one waiting-hall, on one side of which the consulting-rooms are arranged. The seats for patients are grouped opposite the different consulting-rooms. Men sit on the front and women on the back benches. The men are seen first, partly because there usually are fewer male patients, and partly to help them to get back, as soon as possible, to any work that they may be doing. In place of the six rooms formerly available there are now eight rooms. The surgical department now comprises the general consulting-room, separate rooms for surgical dressing for males and females, and a minor operation-room. The medical department comprises a consulting-room and an examining-room, and for each special department there are two rooms available as against the old arrangement of only one. One of the two staircases down into the basement has been entirely removed, its place being taken by a new examining-room, and the patients now all go down one staircase into the basement, where they still pass through the almoner's room and then into the dispensary waiting-room, where, having obtained their medicines, they pass out at one door into the yard, and so through a turnstile into the street. Clear directions for patients are painted on the walls.

A room close to the street and entirely isolated from any part of the building is appropriated to the use of an isolation-room for cases of infectious disease which may happen to be detected in the out-patient department. The walls throughout this department were formerly all bare brick painted, and the partitions enclosing the various rooms were of lath and plaster, and the floors throughout were of deal. The whole of the walls have now been plastered and painted, the lath and plaster partitions removed; the new partitions that have been erected are made of solid plaster slabs, and new seamless flooring has been laid throughout.

### The Mortuary and Post-mortem Rooms.

The mortuary arrangements have been improved. Before the alterations the only access to the Christian mortuary was through the post-mortem room; by removing one exit staircase from the out-patient department the Jews' mortuary has been moved and room has been made for separate entrance to the Christian mortuary, and the very dangerous steps which led up to the post-mortem room and mortuary have been replaced by a sloping way. Both mortuaries and the post-mortem room have been lined with opalite tiles and also refloored. Two new post-mortem tables have been provided.

### Casualty Department.

An alteration of considerable practical value has been made in the arrangement for casualty patients. Formerly the casualty department was on the ground floor of the main building, and consisted of two rooms immediately opposite the Secretary's office. The patients had to be carried up a flight of nine steps and brought in at the front door, a most objectionable and inconvenient arrangement. The casualty department is now in the basement of the ward block, and access to it has been made from the street by a sloping way, which greatly facilitates the carrying of patients in. A small examination-room has been contrived for use in connection with the casualty department out of the space formerly occupied by two w.c's which were not needed. This room is also used as an observation-room for accidents.

### Electrical Department.

The electrical department, which is in the basement of the ward block, has been very considerably improved by throwing in the space formerly occupied by the passage to the various rooms and adding another room for *x*-ray treatment. A glass and iron corridor has been erected outside the basement of the ward block in order to get covered access between the hospital and the chapel, the stores, and the billiard-room. This addition was rendered necessary by the removal of the passage referred to in connection with the electrical department.

### Operation Theatre.

On the third floor of the main entrance block is the operation theatre. Before the alterations the arrangements consisted of one large room, with a portion screened off to serve as an anæsthetising-room, and a room leading out of the theatre, which was used for sterilising, washing, and so forth. The theatre has been reduced in size from 24 feet by 28 feet to 18 feet by 22 feet. The partition formerly enclosing a store-room has been removed and an anæsthetic-room provided, 16 feet by 11 feet, with an entrance from the corridor and a door into the operation theatre itself. The sterilising-room is now approached not directly from the theatre, but through an intervening lobby and a small surgeon's lavatory. The whole of these rooms have been refloored with Terrano; the old cupboards have been cleared out, and the sterilising-room is fitted up with a complete apparatus for steam sterilising. The fireplaces in the theatre and the sterilising-room have been removed, and all these rooms are warmed by hot-water radiators. Ventilation is provided for in the theatre by means of an extract fan, and two screens for filtering the air are provided in connection with the fresh-air inlets behind the radiator. The screens are formed of two thicknesses of thin gauze, "scrim" in teak frames, which slide easily into position. An extra pair of screens is kept prepared with clean "scrim" so that they may readily take the place of the other pair if necessary.

### Hot-water Apparatus.

The only other improvement which need be described at all in detail is that concerned with the hot-water apparatus. The arrangements which formerly obtained were very largely conducive to waste of fuel and inefficiency. A steam boiler in the basement supplied steam to four calorifiers situated in different parts of the building, each calorifier serving a different section of the building. The steam condensed in these calorifiers was allowed to run to waste. The calorifiers are now all grouped in the boiler-house, and the whole of the condensed water is automatically returned to the boilers, and thus provides feed-water at a considerable temperature. There can be no doubt about the saving in the consumption of the fuel effected by this alteration. The amount of warming by hot water has been very considerably increased. In the out-patient department, for example, the whole of the fireplaces have been removed and replaced by radiators. The whole system has been thoroughly overhauled and put into working order, and the net result, as shown by the consumption of fuel up to the present time, is that, notwithstanding the very large additions that have been made to the heating system, there is a definite saving of two tons a week in the consumption of coal, equal to about 40 per cent. of the former consumption.

The Committee view with the utmost satisfaction the architectural skill, ingenuity, and taste displayed; and the minute and careful attention which Messrs. Young and Hall have given to every detail of the work.

## Figures and Tables

**Figure f1:**